# Development and Characterization of Novel Cellulose Composites Obtained in 1-Ethyl-3-methylimidazolium Chloride Used as Drug Delivery Systems

**DOI:** 10.3390/polym13132176

**Published:** 2021-06-30

**Authors:** Iuliana Spiridon, Iuliana-Marilena Andrei, Narcis Anghel, Maria Valentina Dinu, Bianca-Iulia Ciubotaru

**Affiliations:** “Petru Poni” Institute of Macromolecular Chemistry, Grigore Ghica–Vodă 41, 700487 Iasi, Romania; iuliana.marilena.andrei@gmail.com (I.-M.A.); anghel.narcis@icmpp.ro (N.A.); vdinu@icmpp.ro (M.V.D.); ciubotaru.bianca@icmpp.ro (B.-I.C.)

**Keywords:** cellulose, chitosan, polyurethane, lignin, composites, drug delivery, ionic liquids

## Abstract

Two polysaccharides (cellulose and chitosan) and polyurethane dissolved in 1-ethyl-3-methylimidazolium chloride represented the matrix for the obtainment of new composite formulations comprised of lignin, ferrite–lignin hybrid and ketoconazole. The mechanical performances (Young’s modulus and compressive strength) increased with the filler addition. The nature of the filler used in the studied formulations influenced both bioadhesion and mucoadhesion parameters. It was found that the incorporation of lignin and ferrite–lignin hybrid into the matrix has influenced the in vitro rate of ketoconazole release, which is described by the Korsmeyer–Peppas model. All materials exhibited activity against Gram positive (*Staphylococcus aureus* ATCC 25923) and Gram negative (*Escherichia coli* ATCC 25922) bacteria.

## 1. Introduction

Synthesis of multifunctional materials has received much attention in the last decades due to their improved attributes. In spite of their lower properties as compared to synthetic ones, the natural polymers represent one of the most promising options towards a green and sustainable future.

Cellulose and chitin are most abundant natural carbohydrate polymers, having a high degradable and recyclable potential. Cellulose (Cel) is the major component of biomass. It presents excellent mechanical and thermal resistance and is widely used for the production of papers, fibers, textile fabrics, explosives, plastics or composite materials. Cellulose is a linear polymer consisting of carbon, hydrogen and oxygen in the form of β-d-glucopyranose units linked by β-1,4-glycosidic bonds.

Chitosan (CS) and its derivatives are among the promising materials for applications in waste and water treatment, agriculture, cosmetic, food or in medicine [1,2,3] because they are non-toxic, biodegradable and biocompatible polysaccharides [4]. It is composed of 2-glucosamine and N-acetyl-2-glucosamine monomer units and has poor thermal and mechanical properties [5].

Polyurethanes (PU) have numerous applications, such as biomedical, textile, automotive, paintings, adhesives or coatings [6,7], due to their ability to produce well organized and structured materials. Keeping in mind their easy film-forming ability, as well as their enhanced mechanical properties relative to the reinforced composites [8], PU was used as a component of polymeric matrix. The dissolution of cellulose is a challenge, due to its limited solubility in the common solvents. Aiming at 1-ethyl-3-methylimidazolium chloride (EMIC) as an ionic liquid with superior solvent properties, it was used to synthesize a matrix comprised of polysaccharides (cellulose and chitosan) and polyurethane. In this study, we have considered the availability and biocompatible character of polysaccharides, while polyurethane was based due to its contribution in improving the mechanical properties of matrix [9,10].

Lignin (L) is an aromatic polymer consisting of monolignols of substituted phenylpropanoid alcohols. It derives mainly from lignocellulosic biomass that renders its structure directly dependent on the source and delignification method. Lignin exhibits low toxicity and, therefore, the lignin valorization depends on the development of new approaches to overcome challenges that mitigate its conversion relative to potential medical applications [11,12,13]. Organosolv lignin (L) and a cobalt ferrite–lignin hybrid (LH) were added into the Cel-CS-PU matrix in order to evaluate their potential for controlled release applications.

According to P. Figuerido et al. [14], lignin is a promising candidate for drug delivery applications and the superparamagnetic behavior of Fe_3_O_4_-LNPs renders them promising for cancer therapy and diagnosis, such as magnetic targeting and magnetic resonance imaging. The cytotoxicity assays of other authors [15] revealed that lignin coatings can be classified as non-toxic against healthy immunocompetent peripheral blood mononuclear cells. Furthermore, cell studies revealed that alginate–lignin aerogels are non-cytotoxic [16].

Ketoconazole (KK) was selected as a model drug and added into polysaccharides–polyurethane matrix as an antifungal agent, while keeping in mind its recommendations for the treatment of skin, hair and mucosa infections. The resulting material was characterized by mechanical tests and structural investigations. Furthermore, the drug release capacity as well as antimicrobial activity was evaluated.

This work evidences the potential of polysaccharides as matrix for drug delivery and also a promising method to valorize lignin and its hybrids for controlled release applications.

## 2. Materials and Methods

### 2.1. Materials

Cellulose (cotton linters, ~20 micrometers, 240 Da), chitosan, 1-ethyl-3-methylimidazolium chloride and ketoconazole were purchased from Sigma-Aldrich and used without further purification. The polyurethane was synthesized according to Spiridon et al. [17]. Organosolv lignin (extracted with acetic acid/phosphinic acid from birch wood) and its cobalt ferrite hybrid (obtained in our laboratory by combustion of lignin with cobalt nitrate Co(NO_3_)_2_·6H_2_O and ferric nitrate Fe(NO_3_)_3_·9H_2_O at 500 °C) were also added to matrix in order to assess their potential for controlled release applications.

### 2.2. Material Manufacturing

Cellulose (1g), chitosan (0.25 g) and polyurethane (0.25 g) were dissolved in 10 g of 1-ethyl-3-methylimidazolium chloride at 100 °C under stirring for 6 h. Ketoconazole (0.025 g), lignin or cobalt ferrite lignin (0.05 g) were added into Cel-CS-PU matrix and various formulations were obtained (Table 1). Composites were obtained by casting method. After 48 h, the materials were washed with distilled water and dried in a vacuum oven at 40 °C. The IL was recovered by distilling the washed aqueous solution (the IL remained because it is not volatile). The recovered 1-ethyl-3-methylimidazolium chloride was dried under vacuum at 70 °C overnight [18]. The KK was entrapped to a good extent.

The amount of KK released by washing was analyzed spectrophotometrically at λmax value of 254 nm at room temperature. The load KK was calculated according to the following equation:

Load KK = 100 × (m_a_ − m_b_)/m_a_

where the variables are defined as follows: m_a_—initial amount of KK; m_b_—amount of KK found in washings (determined spectrophotometrically based on a calibration curve for KK).

The highest KK load was recorded for Cel-CS-PU-KK (96.84%), followed by Cel-CS-PU-LH-KK (96.12%) and Cel-CS-PU-L-KK (95.65%).

### 2.3. Methods

#### 2.3.1. FTIR

Bruker FTIR Spectrophotometer Vertex 70 (Billerica, MA, USA) equipped with an attenuated total reflection (ATR) device was used to evidence the interactions between components of the composites. All samples were acquired using a diamond crystal with ZnSe focusing element at room temperature.

#### 2.3.2. Mechanical Testing

The mechanical measurements were carried out on swollen samples, at room temperature, using a Shimadzu Testing Machine (EZ-LX/EZ-SX Series, Kyoto, Japan) equipped with a 500 N load cell for compression. The samples were cut into plates with a thickness, width and height of about 10 mm, 10 mm and 3 mm, respectively. A pre-load of 0.1 N was applied before the initiation of each test to ensure complete contact between the sample and the plates of the analyzer. The compression experiments were conducted at a cross-head speed of 1 mm min^−1^. The compressive stress σ (Equation (1)) and the strain ε (Equation (2)) were calculated according to the following equations:

(1) $$\sigma =\frac{A}{F}$$

(2) $$\varepsilon =\frac{\Delta l}{l_{0}}$$

where the variables are defined as follows: *σ* denotes normal stress (N/m^2^); *F* denotes normal force acting perpendicular to the area (N); *A* denotes the area of the plate (m^2^); Δ*l* denotes the change in length (m); *l_o_* denotes initial length (m).

The compressive Young’s modulus of all materials was determined from the slope of the linear part of the stress−strain curves in accordance with the procedure already reported for other bio-based hydrogels. Three different samples were tested for each composition and the values of the modulus of elasticity were expressed as an average value ± SD.

The lyophilized samples were immersed in phosphate buffer saline (PBS) for 24 h to reach saturation prior their testing in a wet state. An initial compressive force of 0.1 N was applied in order to ensure a complete contact between the surface of the plate and that of the sample. The setup of the test and the calculus for the elastic modulus were performed in accordance with the earlier reported procedure [19].

#### 2.3.3. Bioadhesion and Mucoadhesion Properties

The tests were performed using a TA.XT Plus texture analyzer (Stable Micro Systems, Godalming, UK). The maximum force of the detachment and the work of adhesion (the area under the force/distance curve) were determined by using the analyzer software [20]. The material properties were compared with those of the cellulose membrane and stomach tissue. All measurements were performed in triplicates and the adhesion parameters were calculated as mean values with standard deviation.

#### 2.3.4. Scanning Electron Micrography (SEM)

The morphology of materials was evaluated with a scanning electron microscope (SEM—Quanta 200 instrument equipped with an energy-dispersive X-ray spectroscopy (EDX) module, low vacuum mode working) without sputter coating by conducting matter.

#### 2.3.5. In Vitro Release

In vitro release of ketoconazole from all formulations was performed using phosphate buffer saline (PBS) at 37 ± 0.5 °C and pH value of 7.4. Briefly, a weighed amount of materials were placed in a flask with PBS and aliquots of 0.5 mL of the supernatant were removed at different time intervals and diluted to a total volume of 3 mL prior to examination. The amount taken was replaced with fresh PBS in order to maintain the sink conditions. The released ketoconazole was analyzed spectrophotometrically at λ_max_ value of 254 nm at room temperature. The concentrations were estimated based on the calibration curves drawn at the same wavelength. Phosphate buffer saline was used as blank for baseline correction. All tests were performed in triplicate and the results are expressed as mean ± standard deviation.

#### 2.3.6. Evaluation of Antimicrobial Properties

Suspensions were prepared from *Staphylococcus aureus* 25923, *Escherichia coli* 25922 and *Candida albicans* and ATCC 10231 strains in peptone saline with a turbidity of 1° McFarland. A suspension of approximately 1500 UFC (colony-forming units/mL) was obtained by dilution. The surfaces of the tested materials and of the control sample were contaminated with 100 μL ATCC strain (*Staphylococcus aureus* 25923, *Escherichia coli* 25922 and *Candida albicans*). The inoculate was extracted using a sterile swab soaked in peptone saline and has seeded on the surface of the specific environment after 24 h. The plates were incubated at 37 ± 1 °C for 24 h. The colonies were counted and compared to the control sample.

## 3. Results and Discussions

### 3.1. FTIR Spectra

**Figure 1:**
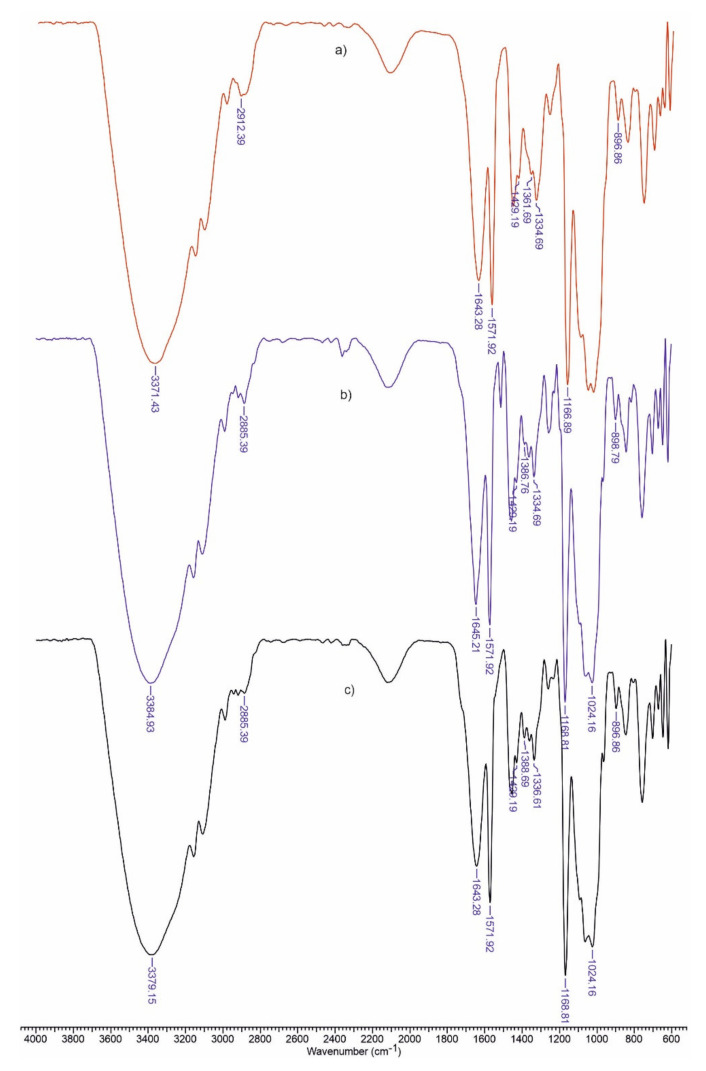
FTIR spectra for Cel-CS-PU (**a**), Cel-CS-PU-L (**b**) and Cel-CS-PU-LH (**c**).

**Figure 2:**
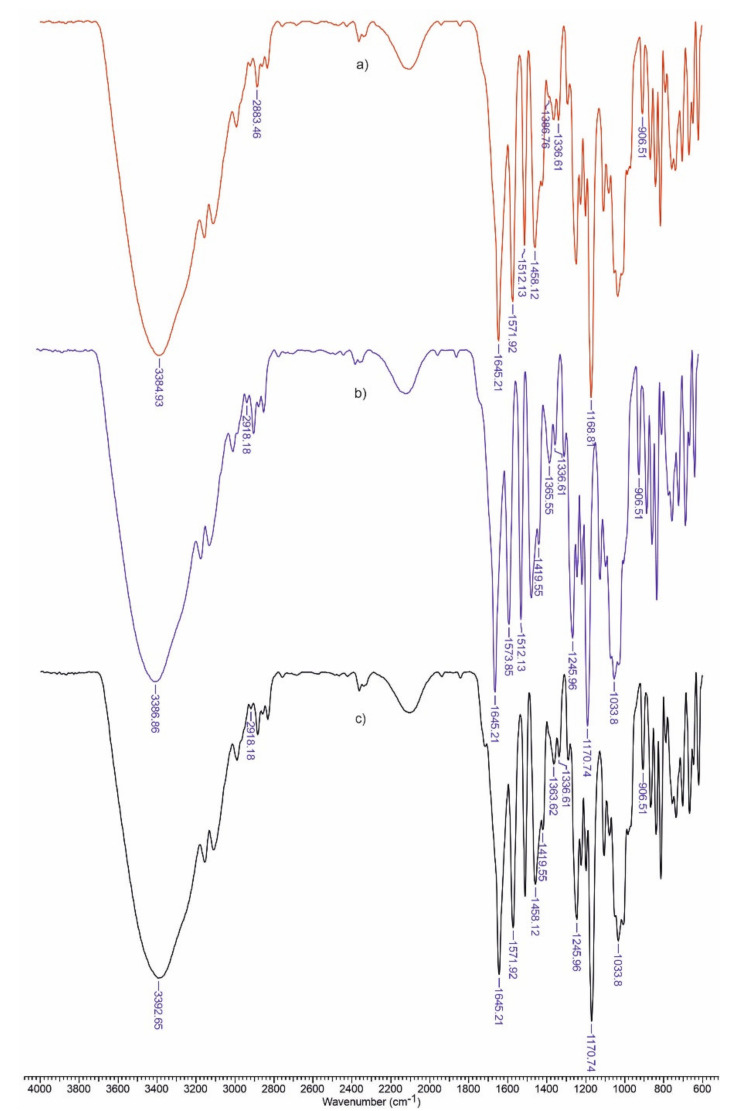
FTIR spectra for Cel-CS-PU-KK (**a**), Cel-CS-PU-L-KK (**b**) and Cel-CS-PU-LH-KK (**c**).

ATR-FTIR spectra (Figure 1 and Figure 2) proved the existence of some interactions between the materials’ components. As expected, the major absorption bands of the components overlap in the case of these biomaterials. We can stipulate the presence of stretching vibrations of hydroxyl groups from 3370 cm^−1^, C–N bond to 1490 cm^−1^, N–H to 1571 cm^−1^ and C=O to 1645 cm^−1^. The signal from 860 cm^−1^ is generally attributed to the C-O-C bonds of the glucopyranose nucleus in polysaccharides.

The spectra are useful for estimating some structural parameters for the tested materials (Table 2). Lateral order index (LOI) was defined as the ratio between the absorption bands at 1437 cm^−1^, which is associated with the amount of the crystalline structure of cellulose and 899 cm^−1^ (β-(1,4) bond in cellulose). The hydrogen bond intensity (HBI) depends upon the crystallinity as well as the amount of bounded water. This index is represented by the absorption ratio at 3400 cm^−1^ (H-bonds between molecules) and 1320 cm^−1^ (CH rocking vibration of the glucose ring).

Total crystalline index (TCI) is calculated as a ratio between absorbances at 1376 and 2902 cm^−1^ [21].

Being an amorphous polymer, lignin’s addition into the matrix decreased the crystallinity degree of the material (TCI). The increase in HBI value by the addition of lignin suggests strong interactions between the matrix components. The addition of ketoconazole decreased the lateral ordering of polymeric matrix.

### 3.2. Mechanical Properties

**Figure 3:**
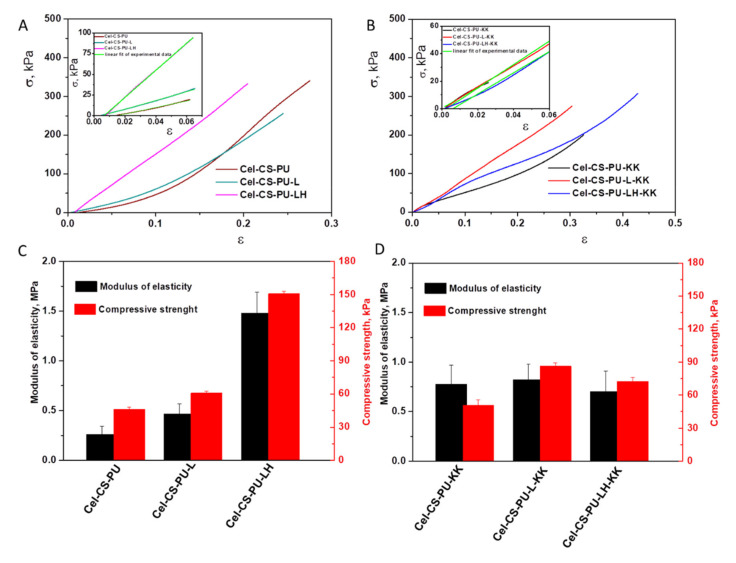
(**A**,**B**) Typical compression stress-strain curves for Cel-CS-PU-based composites obtained by applying a normal force of 100 N under a displacement rate of 1 mm × min^−1^. The insets of Figure 3A,B present the initial linear part of stress-strain curves and the linear fit of experimental data (green lines) from which the compressive Young’s moduli of all samples were calculated. (**C**,**D**). The values of the modulus of elasticity determined for each sample are in agreement with the standard method and the values of compressive strength obtained from the compression stress-strain curves at 10% strain level.

Mechanical behavior is an important factor for the materials that could have potential applications for tissue engineering. Herein, a uniaxial compression test was performed in order to ascertain the effect of the added fillers (lignin, lignin hybrid and/or ketoconazole) on the resulting formulations. The typical stress-strain compression curves are presented in Figure 3A,B. The Young’s modulus and compressive strength increased with the addition of lignin (Cel-CS-PU-L), lignin hybrid (Cel-CS-PU-LH) or ketoconazole (Cel-CS-PU-KK) filler (Figure 3C,D). For instance, the compression strength reaches a maximum of 50.67, 60.71 and 150.8 kPa for Cel-CS-PU-KK, Cel-CS-PU-L and Cel-CS-PU-LH, respectively, at the strain of 10%. The compressive Young’s modulus of these formulations varies from 0.467 to 1.478 MPa. The improvement of the mechanical properties of the filler-loaded Cel-CS-PU materials is well-correlated with the increased cross-linking among components in more hydrogen bonds, which is a fact that is confirmed by the HBI values that are higher for the filler-containing Cel-CS-PU than for neat formulation (Cel-CS-PU without fillers).

Typical compression stress-strain curves for Cel-CS-PU-based composites obtained by applying a normal force of 100 N under a displacement rate of 1 mm × min^−1^. The insets of Figure 3A,B present the initial linear part of stress-strain curves from which the compressive Young’s modulus of all samples were calculated. (C,D) The values of modulus of elasticity determined for each sample were in agreement with the standard method and the values of compressive strength at 10% strain level.

Swelling ratio of all formulations has been evaluated and it is well correlated with mechanical parameters. Swelling ratio displayed the following order: Cel-CS-PU (56.92%) > Cel-CS-PU-L (52.9%) > Cel-CS-PU-LH-KK (48.91%) > Cel-CS-PU-KK (48.03%) > Cel-CS-PU-L-KK (23.99%) > Cel-CS-PU-LH (19.96%).

### 3.3. Scanning Electron Micrography (SEM)

**Figure 4:**
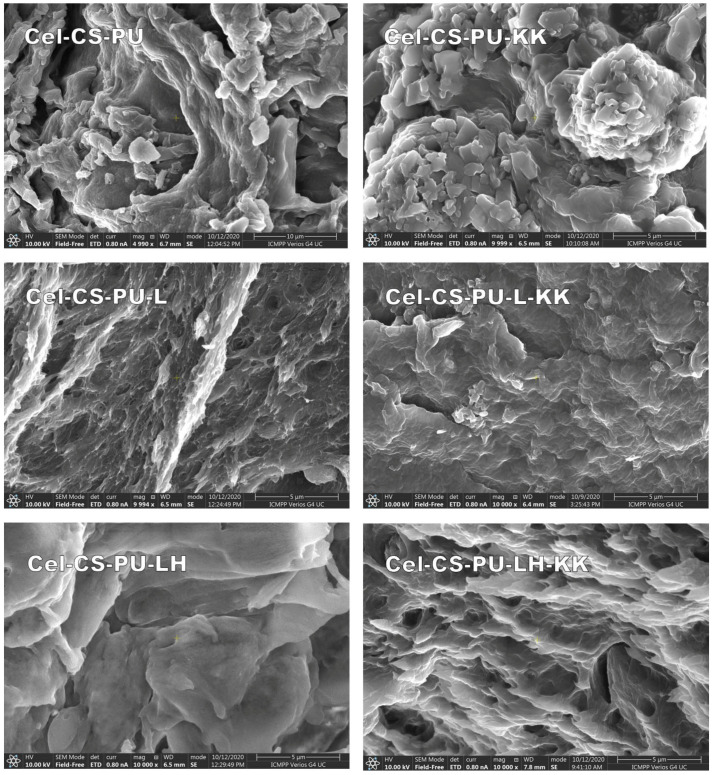
SEM micrographs (×10,000 magnification) of the studied materials.

The morphological features of different formulations are shown in Figure 4. The matrix of composite materials (Cel-CS-PU) presented a rough and porous surface. It seems that presence of lignin and its hybrid increased the degree of binding between components, which is a fact that is confirmed by HBI values.

### 3.4. Bioadhesivity

The nature of the filler used in formulations influenced both bioadhesion and mucoadhesion parameters. In Table 3, the results of bioadhesion and mucoadhesion tests on cellulose dialysis membrane and porcine stomach are presented, respectively. According to these data, the addition of lignin and its hybrid into the Cel-CS-PU matrix reduced the force of detachment and work of bioadhesion. The total work of bioadhesion values is in good agreement with those of adhesive forces. Bioadhesion parameters have increased with KK incorporation. The values of the detachment force indicate stronger interactions with cellulose membrane as compared to those with stomach mucosa.

Mucoadhesion of all formulations was assessed in the stomach at pH 2.6 at 37 °C. The tests performed with porcine stomach tissue evidenced an increase in the force of detachment when lignin and its hybrid was added into Cel-CS-PU matrix, suggesting the ability of matrix components to establish various types interactions (numerous hydrogen bonds and electrostatic interactions) with mucus [22]. A good correlation was observed between the HBI and work of adhesion for Cel-CS-PU-KK, Cel-CS-PU-L-KK and Cel-CS-PU-LH-KK formulations, suggesting that hydrogen bonding may have an important role in the mucoadhesive mechanism between these materials and porcine stomach tissue. Our investigations revealed that Cel-CD-PU-L-KK formulation recorded the highest value for both bioadhesiveness (0.063033N) and mucoadhesiveness (0.0624 N).

Other authors [23] have prepared chitosan based mucoadhesive nanoparticles of ketoconazole and reported an increment in the resistance and release time due to adherence to the stomach mucosa.

### 3.5. In Vitro Release

Due to the fact that the controlled and slow release of drug systems have gained more attention as compared to other drug delivery systems, the evaluation of in vitro drug release performance was carried out in PBS solution to compare the release profile of KK from different formulations. This was affected by the interactions between the materials’ components and the fungal agent, as well as by the diffusion kinetics in the PBS medium. Thus, the drug release rate for the first 200 min was about 15% for Cel-CS-PU-KK and Cel-CS-PU-K-L and about 16.4% for Cel-CS-PU-LH-KK. The same trend was maintained after 500 min (about 49% vs. 56.2%).

**Figure 5:**
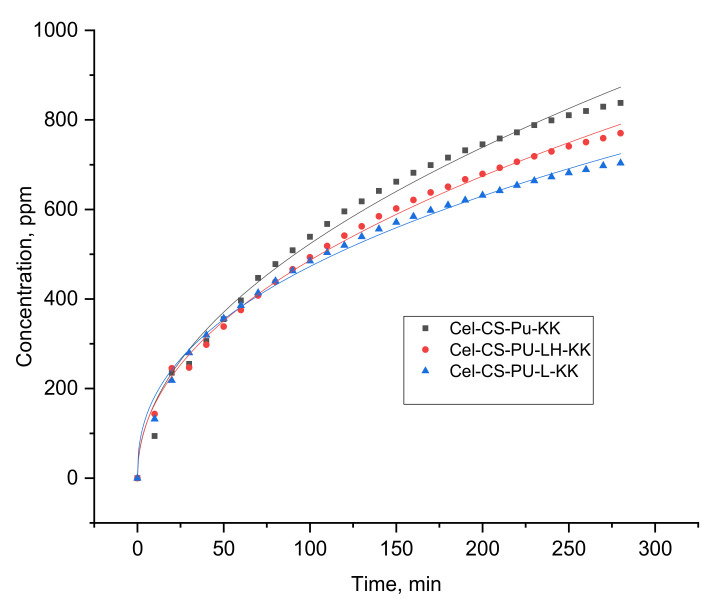
Release profiles of ketoconazole from biocomposites.

According to Figure 5, the presence of organic lignin into polymeric matrix slowly reduced the release capacity of KK, while ferrite–hybrid determined a higher release capacity as compared to that recorded for the Cel-CS-PU-KK. The rapid release of KK from Cel-CS-PU-LH-KK formulation could be explained by the lower BET area of lignin hybrid particles (3.62 m^2^/g) as compared with that of lignin (118.34 m^2^/g).

The Korsmeyer–Peppas model has described the release of KK from all formulations.

The value of *n* (Table 4) was about 0.4 for all formulations (Figure 5 and Table 4) and corresponds to a Fickian diffusion, suggesting that the release mechanisms of ketokonazole were due to its physical diffusion and dissolution as well to the interaction of electrostatic forces or hydrogen bonds [24].

### 3.6. Antimicrobial Activity of the Studied Materials

The capacity of materials for the growth rate inhibition of *E. coli* strain and *S. aureus* strain, which are Gram negative and Gram positive bacteria, was tested. Moreover, *Candida albicans* yeast was considered.

The obtained materials based on Cel-CS-PU matrix have good antimicrobial properties due to the chemical structure of the matrix components that can easily interact with the cell wall of the bacterium. It exhibited the same growth rate inhibition. These results are in good agreement with other similar studies [25] that reported the higher potential antimicrobial activity of hydroxypropylmethylcellulose/chitosan/lignin films against Gram positive bacteria as compared to that exhibited against Gram negative bacteria.

A potentiator effect of ketoconazole on growth inhibition rate has been evidenced on both *E. coli* strain and *S. aureus* (Table 5). The antibacterial activity was more pronounced against *E. coli*, especially for the samples comprising cobalt ferrite—lignin hybrids. This could be due to the damage on the cell membrane caused by ferrite particles. According to Hajipour et al. [26], the cell structure damage and the generation of reactive oxygen species could occur and an improvement of the antibacterial properties is registered. It is worth it to mention that the bactericidal activity of Fe/Co oxide against *S. aureus* and *E.coli* [23] was reported.

The addition of lignin into the polymeric matrix increased the inhibition rate of *C. Albicans*. The material comprised of cobalt ferrite lignin recorded the highest growth inhibition rate (from 65% to 94%). Moreover, *C. Albicans* was more susceptible in comparison with *E. coli* and *S. aureus* after addition of ketoconazole in polymeric matrix.

## 4. Conclusions

Novel composites based on polysaccharides (cellulose and chitosan) and polyurethane dissolved in 1-ethyl-3-methylimidazolium chloride were obtained. Mechanical characterization evidenced that the Young’s modulus and compressive strength increased with the addition of lignin, ferrite–lignin hybrid or ketoconazole. This improvement is well-correlated with the increased cross-linking among components in more hydrogen bonds and confirmed by the HBI values.

The incorporation of lignin and ferrite–lignin hybrid into Cel-CS-PU-KK system has influenced the release profile of KK, which is described by Korsmeyer–Peppas model. The mechanism of drug release was controlled by the Fickian diffusion. Cel-CS-PU-L-KK exhibited the strongest antimicrobial activity, but all materials presented effective inhibition of the bacteria (*Staphylococcus aureus* 25923 and *Escherichia coli* 25922) and yeast (*Candida albicans*). It also recorded the highest value for both bioadhesiveness and mucoadhesiveness.

All obtained data provide information on the suitability of polysaccharides as a matrix for drug delivery and as a promising method to valorize lignin and its hybrids in controlled release applications.

## Figures and Tables

**Table 1:** Various formulations used in the present study.

Code	Component (wt%)					
	Cel	CS	PU	KK	L	LH
Cel-CS-PU	66.6	16.7	16.7	-	-	-
Cel-CS-PU-L	64.5	16.1	16.1	-	3.2	-
Cel-CS-PU-LH	64.5	16.1	16.1	-	-	3.2
Cel-CS-PU- KK	65.6	16.4	16.4	1.6	-	-
Cel-CS-PU-KK-L	63.5	15.8	15.8	1.6	3.3	-
Cel-CS-PU-LH-KK	63.5	15.8	15.8	1.6	-	3.3

**Table 2:** Total Crystalline Index (TCI), Lateral Order Index (LOI) and Hydrogen Bound Intensity (HBI) values obtained from the FTIR spectra analysis of the biomaterials.

Sample	TCI (A_1376_/A_2902_)	LOI (A_1437_/A_899_)	HBI (A_3336_/A_1336_)
Cellulose	1.844	2.174	5.14
Cel-CS-PU	2.349	1.765	3.161
Cel-CS-PU- L	1.159	1.959	4.511
Cel-CS-PU-LH	1.886	1.933	4.685
Cel-CS-PU-KK	1.562	3.093	5.835
Cel-CS-PU-L-KK	1.134	2.523	5.946
Cel-CS-PU-LH-KK	1.554	2.71	6.19

**Table 3:** Adhesive properties of the studied materials.

Sample	Bioadhesivity		Mucoadhesivity	
	Adhesion Force (N)	Work of Adhesion (N × s)	Adhesion Force (N)	Work of Adhesion (N × s)
Cel-CS-PU	0.060433 ± 0.00247	0.005033 ± 0.000231	0.053577 ± 0.005577	0.009333 ± 0.001002
Cel-CS-PU-L	0.046067 ± 0.003435	0.003567 ± 0.000321	0.054833 ± 0.002589	0.0054 ± 0.000624
Cel-CS-PU-LH	0.04508 ± 0.003583	0.0026 ± 0.000721	0.055533 ± 0.003988	0.004433 ± 0.001595
Cel-CS-PU-KK	0.061767 ± 0.002589	0.0062 ± 0.000436	0.055533 ± 0.004561	0.0082 ± 0.000917
Cel-CS-PU-L-KK	0.063033 ± 0.001155	0.0057 ± 0.000361	0.0624 ± 0.00956	0.005733 ± 0.000651
Cel-CS-PU-LH-KK	0.059767 ± 0.00195	0.006333 ± 0.000153	0.050643 ± 0.009454	0.007867 ± 0.001739

**Table 4:** Kinetic parameters for release of ketoconazole from samples.

Samples	*n*	k, min^–n^	R^2^
Cel-CS-PU-KK	0.49	52.6	0.990
Cel-CS-PU-LH-KK	0.47	55.1	0.996
Cel-CS-PU-L-KK	0.41	69.5	0.993

n = release exponent, k = release rate constant, R^2^ = correlation coefficient.

**Table 5:** Antimicrobial activity of obtained composites.

Sample	Growth Rate Inhibition (%)		
	*E. coli* ATCC 25922	*S. aureus* ATTC 25923	*C. albicans*
Cel-CS-PU	97	96	75
Cel-CS-PU-L	98	84	87
Cel-CS-PU-LH	70	83	65
Cel-CS-PU-KK	100	97	100
Cel-CS-PU-L-KK	100	100	100
Cel-CS-PU-LH-KK	93	97	94

## Data Availability

Data sharing not applicable.
